# Early virological response to HIV treatment: can we predict who is likely to experience subsequent treatment failure? Results from an observational cohort study, London, UK

**DOI:** 10.7448/IAS.20.21567

**Published:** 2017-08-30

**Authors:** Nataliya Brima, Fiona C. Lampe, Andrew Copas, Richard Gilson, Ian Williams, Margaret A. Johnson, Andrew N. Phillips, Colette J. Smith

**Affiliations:** ^a^ UCL Institute of Global Health, London, UK; ^b^ Department of HIV Medicine, Royal Free London NHS Foundation Trust, London, UK

**Keywords:** initial virological response, HIV, ART, response to ART, HIV treatment, VL response to ART

## Abstract

**Introduction**: For people living with HIV, the first antiretroviral treatment (ART) regimen offers the best chance for a good virological response. Early identification of those unlikely to respond to first-line ART could enable timely intervention and increase chances of a good initial treatment response. In this study we assess the extent to which the HIV RNA viral load (VL) at 1 and 3 months is predictive of first-line treatment outcome at 6 months.

**Methods**: All previously ART-naive individuals starting ART at two London centres since 2000 with baseline (−180 to 3 days) VL >500 c/mL had a VL measurement between 6 and 12 months after starting ART, and at least one at month 1 (4–60 days) or month 3 (61–120 days) were included. Lack of treatment response was defined as (i) VL >200 copies/mL at 6 months or (ii) VL >200 copies/mL at 6 months or simultaneous switch in drugs from at least two different drug classes before 6 months. The association with VL measurements at 1 and 3 months post-ART; change from pre-ART in these values; and CD4 count measurements at 1 and 3 months were assessed using logistic regression models. The relative fit of the models was compared using the Akaike information criterion (AIC).

**Results**: A total of 198 out of 3258 individuals (6%) experienced lack of treatment response at 6 months (definition i), increasing to 511 (16%) for definition (ii). Those with a 1-month (day 4–60 window) VL of <1000, 1000–9999, 10,000–99,999 and >100,000 copies/ml had a 4%, 8%, 23% and 24% chance, respectively, of subsequently experiencing treatment non-response at 6 months (definition (i)). When considering the 3-month (day 61–120 window) VL, the chances of subsequently experiencing treatment non-response were, respectively, 3%, 25%, 67% and 75%. Results were similar for definition (ii).

**Conclusions**: Whilst 3-month VL provides good discrimination between low and high risk of treatment failure, 1-month VL does not. Presence of a VL >10,000 copies/ml after 3 months of ART is a cutoff above which individuals are at a sufficiently higher risk of non-response that they may be considered for intervention.

## Introduction

For people living with HIV, the first antiretroviral therapy (ART) regimen offers the best chance for a good virological response, which, if achieved, then minimizes the chance of developing resistance [1,2]. Ability to attain a strong initial response, typically defined after six months of ART, is associated with long-term virological suppression. Although in the UK the proportion achieving a good initial virological response to ART is high [1], there is still a small proportion who do not. Earlier identification (within the first three months of ART) of those with a higher probability of not achieving a successful initial response to ART could allow for early interventions to improve the probability that virological suppression will be achieved and prevent the development of resistance.

Several studies conducted to evaluate predictors of virological response to ART in treatment-naive patients have found an association between higher pre-ART HIV RNA viral load (VL) and increased risk of treatment failure by 6–12 months [3–9]. Furthermore, a few studies have evaluated the association between VL levels in the first 1–2 months of ART and subsequent virological response [3,8,9]. However, most of these studies considered the earlier ART calendar period (pre-2000), and results may not be applicable to those starting ART today, with newer, better tolerated or more effective drugs.

This study assesses the ability of the very early VL response to first ART at 1 and 3 months to predict subsequent initial treatment outcome at 6 months in a routine clinical setting among people starting ART from 2000 to 2014.

## Methods

Individuals included in this study were HIV-positive attendees aged ≥18 years at two outpatient clinics in London: the Ian Charleson Day Centre at the Royal Free Hospital and the Mortimer Market Centre. Information was collected from medical notes and electronic records and included demographics, ART treatment history and all VL and CD4 count measurements.

Those included were previously ART-naive and starting an ART regimen consisting of three or more antiretrovirals, including exactly two nucleoside reverse transcriptase inhibitors (NRTIs) and exactly one of a ritonavir-boosted protease inhibitor (PI), non-NRTI (NNRTI), raltegravir or maraviroc between 1 January 2000 and 1 October 2015. In addition, individuals were required to have a baseline VL >500 copies/mL (to ensure previously treated individuals were not included) measured in a window of −180 to +3 days from the start date of the first regimen (“baseline”); a 6 month VL, defined as the first VL between 6 and 12 months after starting ART, and at least one VL measurement at 1 or 3 months. Windows from day 4 to day 60 and from day 61 to day 120 were used, respectively, to define 1 month and 3 month measures. If more than one measurement was recorded in these windows, that closest to 1 and 3 months, respectively, was used.

The outcome of *treatment non-response* was defined in two ways: (i) 6 month VL >200 copies/mL or (ii) either 6 month VL >200 copies/mL or simultaneous switch/stop of antiretrovirals of at least two drug classes by 6 months. A cutoff of 200 copies/ml was used as low-level blips likely do not constitute treatment failure. The inclusion of treatment switch/stop was intended to capture situations where treatment was changed due to perceived virological failure prior to 6 months. Switch of drugs from two or more classes was required to exclude switches made solely for toxicity reasons. Only switches and stops made 28 days to 6 months after starting ART were included.

The unadjusted and adjusted associations of VL, change in VL from baseline and CD4 count, at months 1 and 3 (predictor variables), with treatment response at 6 months (outcome variable) was assessed graphically and using logistic regression. Current VL was considered as a categorical variable (<200, 200–499, 500–999, 1000–9999, 10,000–99,999 and ≥100,000 copies/ml) and as a continuous variable on the logarithmic_10_ scale. Multivariable analyses were adjusted for age (continuous), ethnicity (white, black or other), gender/likely HIV acquisition route (men who have sex with men, men who have sex with women, women) and treatment type (1 NNRTI+2 NRTI, other). To compare the predictive ability (goodness-of-fit) between the different measures, the Akaike Information Criterion (AIC) for the models were calculated [10] restricting analysis to attendees with all measures recorded, a lower value indicates better fit.

Two sensitivity analyses were performed. First, treatment non-response was re-defined changing the cutoff from 200 to 50 copies/ml. Second, analyses were repeated on the subset who started ART in the later years of 2008–2014.

All data analyses were performed using STATA version SE13. The Royal Free HIV Cohort and the Mortimer Market HIV cohort have received favourable ethical review by the Royal Free Hospital and Medical School, and the Camden and Islington NHS Research Ethics Committees. Individual participant consent was not required on the premise that the analysis was conducted on an anonymized data set. The study did not receive external funding.

## Results and discussion

Of 6059 individuals starting ART between 1 January 2000 and 30 October 2015, 1366 (22%) had a missing pre-ART VL, 536 (9%) had a baseline VL ≤500 copies/ml, 539 (9%) did not have a VL measurement at 6 months post-ART and 360 (6%) did not have a VL measured at either 1 or 3 months post-ART. Therefore, 3258 individuals (54% of all those recorded as starting ART) were included; 2938 (90%) and 2223 (68%) had VL measurements after 1 and 3 months of ART, respectively; 1903 (58%) had a measurement at both time points.

Most individuals were men (2583; 79%), of white ethnicity (2021; 62%), and 64% (2080) were men who likely acquired HIV through sex with other men. At ART initiation, median age was 38 years [interquartile range (IQR) 33–44], median CD4 cell count was 222 cells/mm^3^ (124–321) and median VL was 4.95 log_10_ copies/ml (4.43–5.41). Most individuals (1985; 61%) started 1 NNRTI+2 NRTI, 37% (1210) started 1 PI/r + 2 NRTI and 2% (63) started 1 integrase inhibitor + 2 NRTI. The most frequently used drug regimens were efavirenz+tenofovir+emtricitabine (932; 29%), efavirenz+lamivudine+zidovudine (319; 10%) and lopinavir/ritonavir+lamivudine+zidovudine (177; 5%).

A total of 198 individuals (6%) had a VL >200 copies/ml after 6 months of ART, and so experienced a lack of treatment response using definition (i). An additional 313 individuals stopped or switched antiretroviral (ARV) drugs from at least two drug classes (median [IQR] time to stop/switch: 105 [57–149] days), leading to 16% (511) experiencing a lack of treatment response using definition (ii). A total of 39 individuals experienced both VL >200 copies/ml and made an eligible ARV change. Of the individuals with lack of treatment response according to definitions (i) and (ii), 84 out of 198 (42%) and 270 out of 511 (53%), respectively, had achieved VL <200 copies/mL at month1 and/or at month 3.

**Figure 1.**
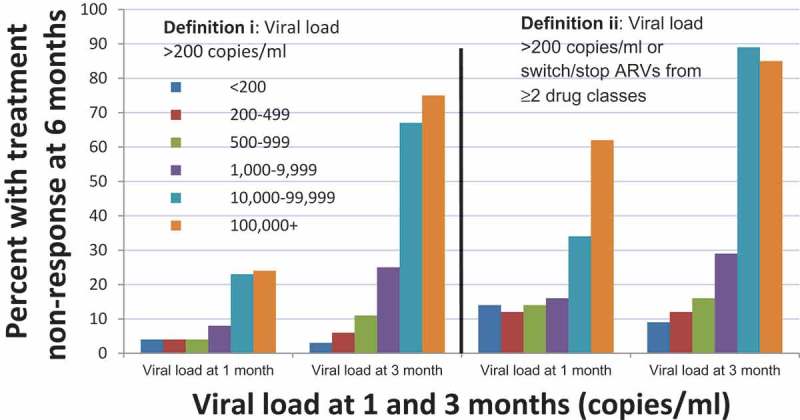
Per cent with HIV treatment non-response after 6 months of antiretroviral therapy, according to HIV RNA viral load measured 1 month 3 months after the start of ART.

Of those who achieved VL <200 copies/ml on their 1 month VL value, only 4% did not have a treatment response at 6 months using definition (i) (Figure 1),

compared to 8%, 23% and 24% among those with a 1 month VL of 1000–9999; 10,000–99,999 and ≥100,000 copies/ml, respectively (*p* < 0.001; chi-squared test). When considering definition (ii), these figures were 14%, 16%, 34% and 62%, respectively (*p* < 0.001). Similar trends were seen for the 3-month VL, with even greater differences between groups in the probability of non-response. The figures were 3%, 25%, 67%, 75% for definition (i) and 9%, 29%, 89%, 85% for definition (ii), respectively (*p* < 0.001). For both the 1- and 3-month time points, and for both endpoint definitions, the risk of treatment non-response substantially increased at a VL cutoff of 10,000 copies/ml, with a further increase above 100,000 copies/ml for definition (ii).

Individuals with a VL ≥10,000 copies/ml after 1 month of ART had 5.7 times the odds (OR = 5.7; 95% CI 3.6–9.0) of treatment non-response by 6 months (definition (i)) compared to those who had VL <10,000 copies/ml, and 4.2 times the odds using definition (ii) (OR = 4.2; 2.90–6.15), see Table. After 3 months of ART, individuals with a VL ≥10,000 copies/ml had almost 51.2 times the odds (OR = 51.2; 27.9–93.8) under definition (i) and 60-fold increased odds using definition (ii) (OR = 59.8; 26.8–133.6) to have treatment non-response at 6 months.

To assess the ability to predict treatment response, the AIC was calculated based on logistic regression models in which each measure (VL, change in VL) was modelled as a continuous rather than a categorical variable (Table 1).

**Table 1.** Ability of early viral loads to predict 6-month ART response

	Definition (i): VL >200 copies/ml					Definition (ii): VL >200 copies/ml or switch/stop ARVs from ≥2 drug classes				
	Non-response *n* = 198 (6%)	Responder *n* = 3060	^1^OR (95% CI)	^2^Adjusted OR (95% CI)	^3^AIC, Univariate/adjusted model	Non-response *n* = 511 (17%)	Responder *n* = 2747	^1^OR (95% CI)	^2^Adjusted OR (95% CI)	^3^AIC Univariate/adjusted model
**Viral load (log_10_ copies/ml), Mean (SD)**										
pre-ART	4.97 (0.91)	4.88 (0.75)				4.86 (0.84)	4.89 (0.74)			
1 month	3.05 (1.01)	2.60 (0.72)	*p* < 0.001 1.90 (1.57–2.29)	*p* < 0.001 1.96 (1.62–2.38)	1129/1086	2.83 (0.96)	2.59 (0.69)	*p* < 0.001 1.44 (1.26–1.65)	*p* < 0.001 1.62 (1.41–1.86)	2239/2185
3 months	3.03 (1.39)	1.92 (0.45)	*p* < 0.001 3.96 (3.22–4.87)	*p* < 0.001 4.05 (2.26–5.03)	740/713	2.54 (1.21)	1.91 (0.41)	*p* < 0.001 3.08 (2.56–3.70)	*p* < 0.001 3.30 (2.71–4.01)	1352/1330
**Decrease in viral load from pre-ART value (log_10_ copies/ml), Mean (SD)**										
Change at 1 month	1.95 (0.96)	2.27 (0.71)	*p* < 0.001 0.59 (0.48–0.71)	*p* < 0.001 0.62 (0.51–0.76)	1144/1111	2.05 (0.91)	2.29 (0.69)	*p* < 0.001 0.67 (0.58–0.77)	*p* < 0.001 0.63 (0.55–0.73)	2235/2191
Change at 3 months	2.00 (1.44)	3.00 (0.77)	*p* < 0.001 0.36 (0.30–0.43)	*p* < 0.001 0.38 (0.32–0.46)	804/788	2.37 (1.28)	3.02 (0.74)	*p* < 0.001 0.46 (0.40–0.53)	*p* < 0.001 0.46 (0.39–0.53)	1400/1388
**CD4 count (cells/mm^3^), Mean (SD)**										
Pre-ART	225 (189)	245 (170)				225 (189)	244 (170)			
1 month	307 (204)	336 (193)	*p* = 0.033 0.97 (0.94–1.00)	*p* = 0.736 0.99 (0.96–1.03)	1166/1130	326 (188)	336 (195)	*p* = 0.218 0.99 (0.97–1.01)	*p* = 0.991 1.00 (0.98–1.02)	2265/2230
3 months	341 (221)	367 (198)	*p* = 0.007 0.95 (0.92–0.99)	*p* = 0.342 0.98 (0.95–1.02)	923/897	358 (214)	367 (197)	*p* = 0.104 0.98 (0.96–1.00)	*p* = 0.651 0.99 (0.97–1.02)	1513/1503

VL = viral load; ART = antiretroviral therapy; AIC = Akaike information criterion; the lower the value of the AIC, the better model fit.

^1,2^Main predictors VLs at 1 and 3 month fitted on logarithmic scale (see methods).

^2^Adjusted for age (continuous), ethnicity and gender/likely risk for HIV acquisition, and treatment regimen.

^3^To calculate AIC analysis was restricted, separately for analysis of 1 and 3 month measures, to patients who had VL, pre-ART VL and CD4 count recorded.

The results indicate that for either definition of response, and for univariate or multivariate models, the 3 month VL has the best predictive ability of the measures considered. Conversely, CD4 count at 1 and 3 months did not predict non-response in multivariate models. In addition, men who likely acquired HIV heterosexually (adjusted odds ratio [aOR] = 2.69 vs. men who have sex with men CI 1.44–5.03, *p* = 0.002); women (aOR = 1.97, CI 1.02–3.81; *p* = 0.044) and those of black ethnicity (aOR = 1.85 vs. white; CI 1.01–3.40, *p* = 0.046) had higher odds of treatment failure at six months using definition (i). Increasing age (aOR = 0.98 per 1 year older; 95% CI 0.96–1.01; *p* = 0.127), ethnicity others OR = 1.24, CI 0.62–2.57; *p* = 0.545 and those on an NNRTI-containing regimen (aOR = 1.05 vs. other; CI 0.69–1.59; *p* = 0.835) were not significantly different.

The results of all sensitivity analyses were consistent with the main findings.

Although the proportion of individuals starting ART in the period 2000–2014 and not achieving an initial virologic response was small, we identified subgroups at greater risk. We found that most individuals with a VL >10,000 copies/ml after 3 months of ART did not achieve viral suppression at 6 months and their risk was much greater than those with lower VL. A VL >10,000 copies/ml at 3 months seems a reasonable criterion to identify individuals for early intervention to increase the effectiveness of treatment. A VL >10,000 copies/mL at one month is a weak early warning sign that viral suppression will not be achieved. While most people in this situation do still achieve suppression at 6 months, this may be, at least in part, because clinics are already starting to emphasize good ART adherence and helping to address any identified issues.

Besides showing that the best predictor of 6 month treatment non-response is a VL at 3 months and that one month VL is a less strong predictor, our results also suggest that current VL levels are superior predictors to change in VL from pre-ART levels. In other words, we suggest the current VL measure is more important than considering the speed of VL decline. Finally, the CD4 count had little predictive ability over this early treatment period.

The considerably higher treatment non-response rates seen in the highest 1 and 3 month VL categories under definition (ii) compared to definition (i) suggests that clinicians are already successfully intervening in this group through treatment changes before the 6-month time point was reached. These early VL results are reported to treating clinicians, who may be interpreting them as an early measure of adherence and intervene accordingly. Nonetheless, the large effect sizes seen in this study suggest that further intervention could be possible.

In previous studies, baseline VL has been shown to be associated with the probability of achieving VL <500 copies/ml [5–9]. Early VL measures have previously been shown to be a good predictor of treatment failure by 6–12 months [3,4]. Furthermore, a handful of studies have looked at relationships between the early viral response to ART and longer-term viral response results after 6–12 months of ART [4,7–9]. Cozzi Lepri and colleagues in 2001 [9] showed that 4 and 8 week VLs are strong predictors of viral response over the first 24 weeks of ART in a clinical cohort of patients starting ART. Raboud and colleagues [4] found that VL measurement at 16 weeks is predictive of virological success at 1 year of triple therapy among patients in a clinical trial. However, these studies were carried out pre-2000. In recent years, more effective and better tolerated antiretroviral drugs have become available. Integrase inhibitors have been shown to have a very potent and rapid antiretroviral effect [11]; therefore, the usefulness of early VL measures to predict long-term virological outcome may have changed for people starting ART more recently.

In our analysis, we only included those with a sufficient number of VL measurements. Therefore, we may have excluded infrequent clinic attenders or those lost to follow-up, for whom levels of treatment response may be poorer. We did not have data for all participants on the reasons for switching or stopping in our data set and so we attempted to capture stopping due to perceived treatment failure by using a definition that incorporated major changes to the ART regimen. Our sample included only a small number of people starting integrase inhibitors which are now recommended as part of first-line regimens [12], and so we were unable to examine whether associations differed in this subgroup. Although we have constructed a definition of switch for failure based on advice from treating clinicians at the two participating centres, it is possible that some of the switches included under the definition (ii) of lack of treatment response were for toxicity reasons, rather than virological failure. We did not have data on transmitted drug resistance, which is currently estimated to have a prevalence of 7% in the UK [13]. This may have been an important factor among those who never achieved virological suppression. Finally, we also did not have information on participants’ adherence.

## Conclusions

Very early VL measures taken 1 and 3 months after the start of ART could be a useful indicator of initial treatment non-response. We suggest that a value >10,000 copies/ml at 3 months identifies individuals at a high risk of non-response at 6 months and should prompt specific interventions, such as initiating adherence support strategies. This criterion could also be used to select a population to test candidate interventions, such as addition of an agent from another class of ART.
